# Supplementary material for: Patterns of Intron Gain and Loss in Fungi
Source: PLoS Biol. 2004 Nov 30;2(12):e422. doi: 10.1371/journal.pbio.0020422 (PMC532390; doi:10.1371/journal.pbio.0020422)
Supplement: Table S1 — Also available at http://genes.mit.edu/NielsenEtAl/. (4.3 MB ZIP). [file pbio.0020422.st001.zip › NielsenEtAl/html/1428.html]

AN8274.1.NCU01689.1.MG07201.1.FG01421.1


```
 CLUSTAL W (1.82) Multiple Sequence Alignments - Introns Inserted


Sequence 1: NCU01689.1	316 aa
Sequence 2: MG07201.1	317 aa
Sequence 3: FG01421.1	317 aa
Sequence 4: AN8274.1	350 aa
Alignment Length: 354 aa
Number Identitical Residues: 220 aa
Alignment Score (without introns) 9360


MG07201.1 	MSVAVPPGSPAPT-LEKLEKKPVKFSNLLL~-------GAGLNMFE~VTTLGQ~PLEVVK
NCU01689.1	MSAAVAS-VPAPA-PKKLEKKPIKFSNLLL~-------GAGLNLFE~VTTLGQ~PLEVIK
FG01421.1 	-MATLRTDLPGPIGDKKLEKKPIKFSNLLL~-------GAGLNLFE~VTTLGQ0PLEVVK
AN8274.1  	MSTATEELGTAPK----LEKKPVKFSNLLC1SDAITTVGAGLNLFE2VTTLGQ0PLEVIK
          	  .:     ..*     *****:******  :.: :: *****:** ****** ****:*

MG07201.1 	TTMAANRGDGFTTALGRIWGRGGVLGF1-----------------------------YQG
NCU01689.1	TTMAANRNDGFTSALRRIWNRGGIFGF1-----------------------------YQG
FG01421.1 	TTMAANRGDSMAKSLGRVWARGGPLGF1-----------------------------YQG
AN8274.1  	TTMAANRGDSFAGAMGRIWGRGGILGC~KYTRNPHTRISRGMGWVNIFGLTRVCRKDYQG
          	*******.*.:: :: *:* *** :*  . : .. :  : . .  .  . :  . ..***

MG07201.1 	LIPWAWIEASTKGAVLLFVASEAEYYARSYGASEFGGGIAGGVTGGVAQAYATMGFCTCM
NCU01689.1	LIPWAWIEGSTKGAVLLFVASEAEYHARAAGAGDFVAGITGGIVGGVAQAYATMGFCTCM
FG01421.1 	LIPWAWIEASTKGAVLLFVASEAEYYARAAGASEFGGGILGGVTGGVAQAYATMGFCTCM
AN8274.1  	LIPWAWIEASTKGAVLLFVASEAEFRAKVLGAPDFLAGISGGMAGGIAQAYATMGFCTCM
          	********.***************: *:  ** :* .** **:.**:*************

MG07201.1 	KTVEITKHKMAATGQAPQGTWATFMDIYRREGIRGINKGVNAVAIRQMTNWGSRFGLSRL
NCU01689.1	KTVEITKHKMSAAGQKAPGTWATFMDIYRREGIRGINKGVNAVAIRQMTNWGSRFGLSRL
FG01421.1 	KTVEITKHKLAASGVKPQSTFQTFGEIYRKEGIRGINKGVNAVAIRQMTNWGSRFGLSRL
AN8274.1  	KTVEITKHKMAAQGVKPPSTFATFMDIYRKEGIRGINRGVNAVAIRQTTNWGSRFGLSRL
          	*********::* *  . .*: ** :***:*******:********* ************

MG07201.1 	AEQGIRDVTGKKHGEKLSAWEKILASSLGGGLSAWNQPIEVIRVEMQSKKDDPNRPKKMT
NCU01689.1	AEQGIRKATGKEEGQKLAAWEKILASALGGGLSAWNQPIEVIRVEMQSKKEDPNRPKKMT
FG01421.1 	AEGWMKSLTGKKEHEKLSAGEKVIASALGGGLSAWNQPIEVIRVEMQSKKEDPNRPKKMT
AN8274.1  	AEQALRKVTGKDESQKLSALEKVIASGLGGGLSAWNQPIEVIRVEMQSKTEDPNRPKNLT
          	**  ::. ***.. :**:* **::**.**********************.:******::*

MG07201.1 	VGNTFKYIYQTNGIKGLYRGVAPRIGLGVWQTVCMVALGDM2AKTYVEKVTGEKVTAKH
NCU01689.1	VGNTFRYIYQTNGIKGLYRGVTPRIGLSIWQTVCMVAFGDM2AKTYVEKLTGDAVTAKH
FG01421.1 	VGNTFKYIYESNGIKGLYRGIAPRISLGVWQTVCMVAFGDM2AKAYVEKVTGEAVTAKH
AN8274.1  	VGKAFKYIYDTNGIKGLYRGVTPRVGLGIWQTVCMVALGDM2AKEAVEKLTGDKVTAKH
          	**::*:***::*********::**:.*.:********:*** **  ***:**: *****
```
